# A cranio-encephalic trauma due to electric-scooter accident: could the wearing of a helmet reduce this risk?

**DOI:** 10.1007/s12024-022-00477-2

**Published:** 2022-05-13

**Authors:** Giovanni Aulino, Matteo Polacco, Vincenzo Fattoruso, Francesca Cittadini

**Affiliations:** Department of Health Surveillance and Bioethics, Fondazione Policlinico A. Gemelli IRCCS, Università Cattolica del Sacro Cuore Largo F. Vito, 00168 Rome, Italy

**Keywords:** Electric scooter accidents, Cranio-encephalic trauma, Forensic pathology, Helmets

## Abstract

Nowadays, one of the most important health and social policy issues concerning all countries is the problem of road accident rates. Traffic is one of the most important risk factors. For this reason, ridesharing companies have been launching electric scooters in Rome since June 2019 with the aim of reducing car traffic. In the absence of relevant legislations, the risk is that of facing an increase in deaths due to electric scooter crashes. We report the case of an electric scooter accident victim with cranio-encephalic trauma associated with limb injuries that caused immediate death. This case report emphasizes how the obligation of using helmets must be extended to all ages, in order to reduce the risk of increasing the number of deaths. Compulsory helmet use can reduce fatalities in all cases where high-speed crashes are not involved.

## Case report

During the night, a 33-year-old man was driving a shared electric-scooter when it collided with another vehicle. He suffered significant injuries and was pronounced dead after half an hour.

The event was reconstructed, and a frontal collision between the car and the e-scooter was determined to be the most likely scenario. The patient’s right leg was the first to be struck in the accident with the automobile. The man then hit his head against the windshield before falling to the ground, according to the dynamics.

Autopsy and toxicological analyses were conducted.

He had a body mass index of 27.2 (height 167 cm and weight 76 kg).

External examination revealed injuries to the head, chest, and extremities.

**Fig. 1:**
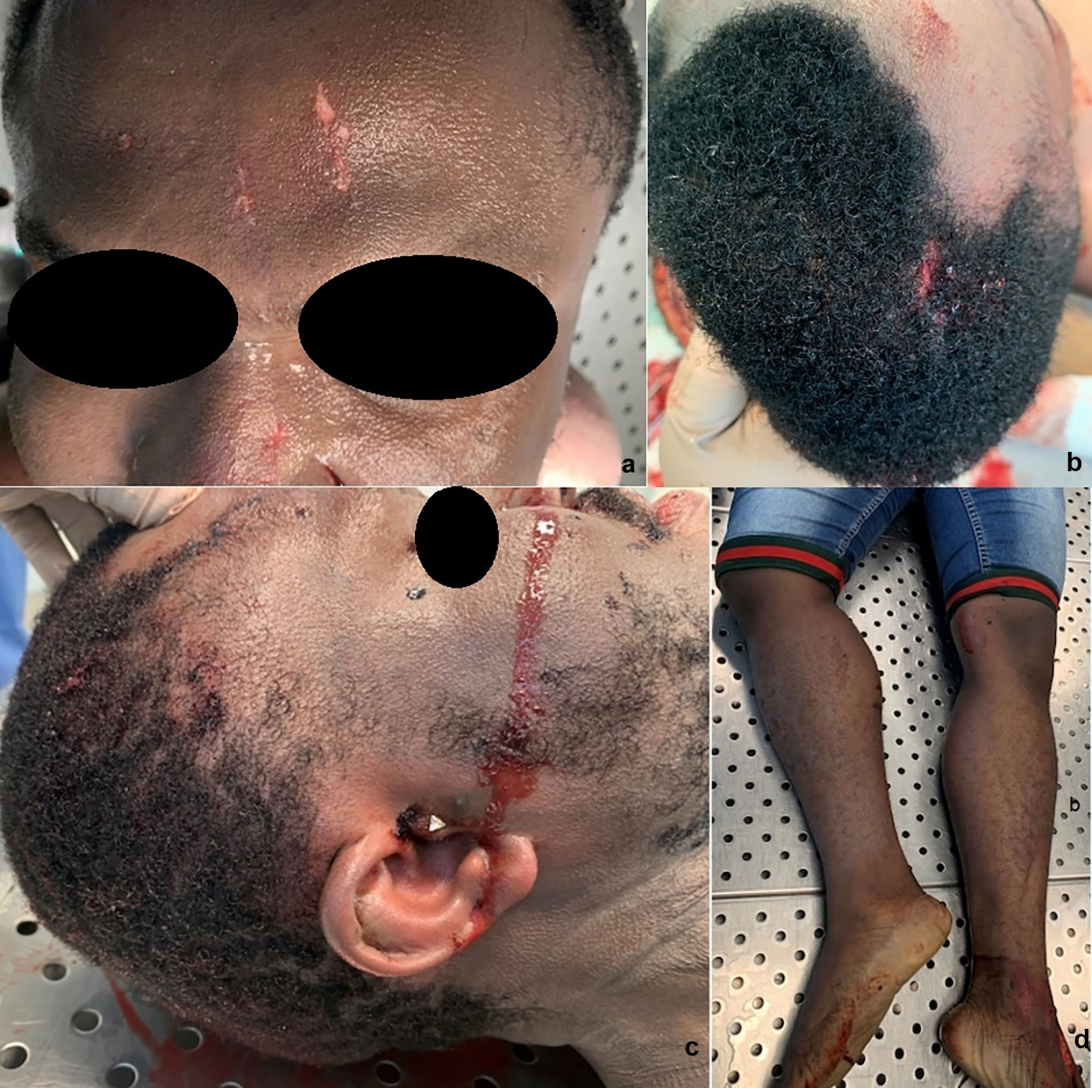
External examination. **a** Two abrasions in the frontal region.** b** 1-cm lacerated wound in right front-parietal region. **c** Blood-serum material from the nostrils. **d** Right lower limb extra-rotation

On head inspection, despite hitting it on the windshield, only two abrasions in the frontal region (Fig. 1a), a 1-cm lacerated wound in the right front-parietal region (Fig. 1b) and leakage of blood-serum material from the nostrils (Fig. 1c) were observed.

Multiple abrasions between the IV and IX ribs, with a vertical axis and dimensions of 12.5 cm × 9.5 cm, were reported at the level of the thorax.

A 3.5-cm-wide triangular lacerated wound in the left upper limb’s olecranon region was observed.

The right lower limb was extra-rotated and shortened (Fig. 1d), and a 3.5-cm-wide irregular excoriation on the right knee was detected.

Palpation and passive mobilization revealed preternatural mobility in the upper third of the right thigh, indicating a femur fracture.

On inspection of the lower limb, a 5-cm-long lacerated wound in the posterior portion of the left foot and a 1-cm-long wound positioned medially with respect the former were observed.

**Fig. 2:**
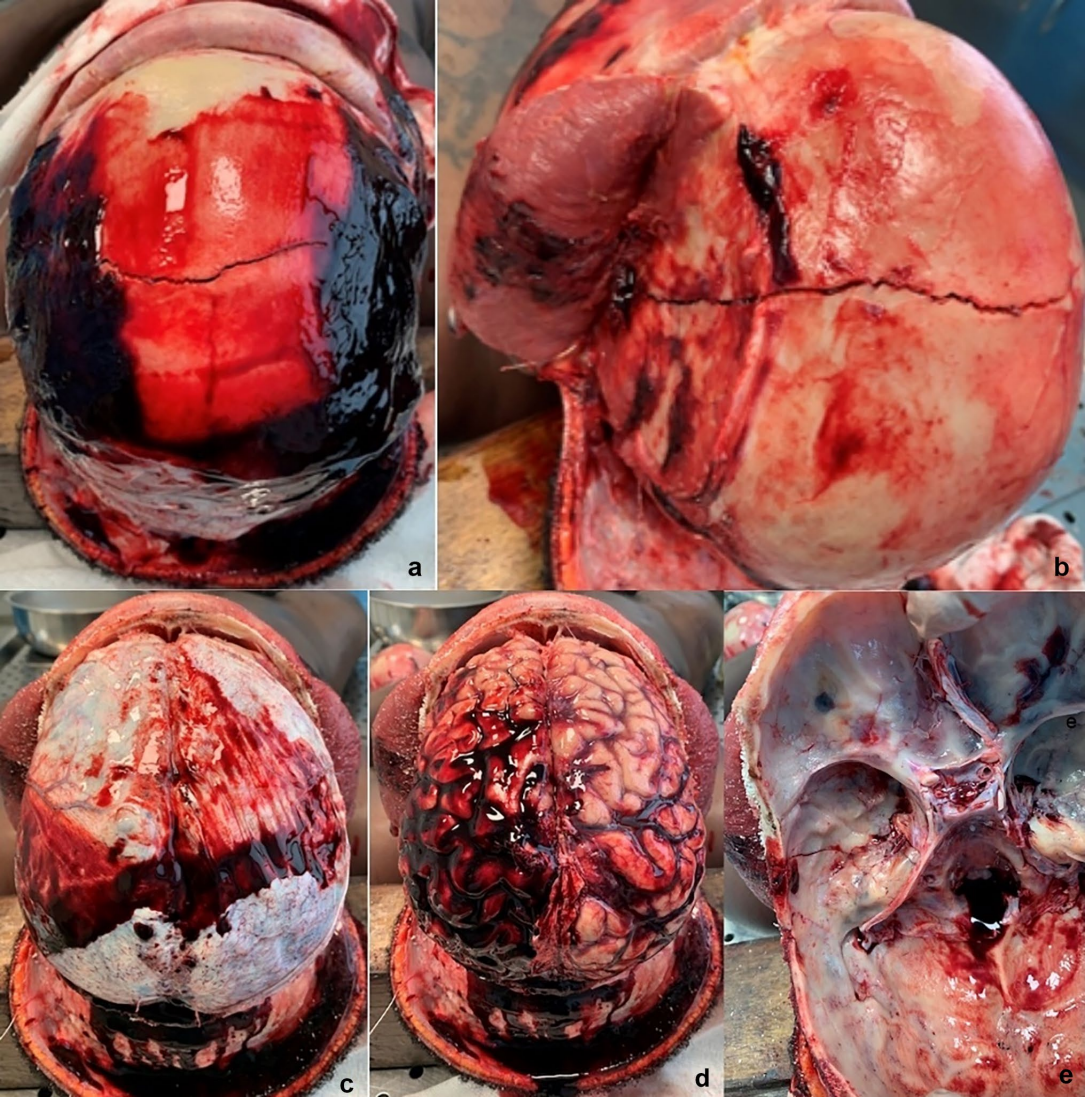
Head internal examination. **a** Diffuse hematic infiltration of galea capitis and periosteum more marked in the bi-parietal region, as well as infiltration of the bundles of the temporal muscle. **b** Fracture with a coronal course from right parietal region to left temporal region. **c** Blood painting of dura mater. **d** Sub-dural hemorrhage especially in cerebral hemisphere. **e** Continuation of the fracture into cranial base

In addition, autopsy demonstrated a galea capitis and periosteum hematic infiltration (Fig. 2a), mainly at the level of the bi-parietal region and left temporal muscle. The vault of the skull presented a long fracture, with minimal margins’ diastasis, which displayed a coronal course and extended from the right parietal region to the left temporal region (Fig. 2b).

A dura mater painted with blood (Fig. 2c) and a sub-dural hemorrhage at the level of the left cerebral hemisphere were detected (Fig. 2d). Once the brain was removed, it was discovered that the fracture of the vault of the skull continued up to the left petrous rock at the level of the cranial base, and a hairline fracture with coronal course on the right orbital roof (Fig. 2e).

The brain (1620 g) appeared congested and edematous, with modestly flattened circumvolutions, and subarachnoid hemorrhage, mostly in the bi-parietal and cerebellar areas. The ventricles contained cerebrospinal fluid stained with blood. On section of cerebellum and brainstem, lacerations foci were appreciated on the posterior surface of the left cerebellar hemisphere. The presence of blood was observed at the level of the ponto-cerebellar cistern.

Neither injuries nor fractures were observed at the internal examination of both thorax and abdomen.

Soft-tissue infiltrations were observed adjacent to the displaced right femur fracture.

Toxicological analyses were negative for both substances and alcohol.

## Discussion

In Italy, a trial of electric scooter circulation began in June 2019 aimed at reducing pollution [1]. Due to the pandemic, it was not possible to observe the consequences of the introduction of these vehicles during 2020, despite recent studies highlighting an increase in injuries and admissions associated with electric scooter (e-scooter) use in several countries [2–4].

In the USA, e-scooter-related injuries have increased in concomitance with the increase in their use. Analysis of the National Electronic Injury Surveillance System from 2014 to 2018 demonstrated a 222% increase in‐scooter injury incidence and a 365% increase in hospital admission, with the steepest rises occurring from 2017 to 2018 [5].

In a retrospective case series, 52 out of 90 patients displayed injuries of the head and face; of these, 58% were considered severe (i.e., fracture, internal hemorrhage, concussion, loss of consciousness) [6].

Most electric scooter-related injuries involve a single user, with falls being the most common mechanism of injury [7].

To our knowledge, this is the first case report analyzing the causes of death in an electric scooter accident; the most injured body parts were the head, upper extremities, and lower extremities as expected. This injury pattern is like that observed in certain non-motorized mobility devices such as skateboards and non-motorized scooters [8].

However, e-scooter injuries are more likely to be severe due to their increased speeds [9].

In the current case, the cause of death was craniocerebral trauma characterized by a fracture of the cranial vault and the skull base, an intraparenchymal hemorrhage, and a cerebellar lacerative-concussive focus.

Low rates of helmet use among riders were noted, which may be linked to the high prevalence of head injuries following e-scooter-associated trauma. Moreover, one study noted a protective effect of helmets on craniofacial injuries suggesting many of these injuries may be preventable [10].

In Italy, there are no studies demonstrating a reduction in crash mortality with e-scooters: indeed, compulsory helmet use for motorcyclists has led to a 40% reduction in fatalities [11].

In addition, although due to the pandemic it was not possible to study accident trends in e-scooters, in 2019, there was an increase in accidents due to increased e-bikes circulation [12].

However, e-scooters are a one of the best choices for reducing pollution in major metropolitan areas like Rome; as a result, the circulation of these vehicles should be supported on the one hand, while prevention campaigns should be implemented on the other.

Maximum speed reduction is not enough: in our case, the speed was most likely not high, even though the impact resulted in severe craniofacial injuries.

Compulsory helmet use and access to well-regulated bicycle lanes can reduce fatalities in all cases where high-speed crashes are not involved.

The scooter sharing companies, for example, should provide a box in which to keep the helmet to encourage use.

In Italy, although some limitations have been introduced by legislation, helmets are only compulsory for people under 14 years old [13].

In conclusion, we believe that this case can serve as an example of how forensic pathology knowledge and research can be applied to the community.

Indeed, the critical approach that is characteristic of forensic pathology helps in detecting new risks for the community; in particular, in the case at hand, it allowed us to emphasize the need to extend the helmet obligation to all ages, in order to reduce the number of e-scooter-related deaths and injuries. These prevention strategies could lend a hand in increasingly encouraging the use of *‘*zero-impact’ transportation which is an innovative and economically advantageous method of urban transport.

## Data Availability

My manuscript has associated data in a data repository.
